# Categorizing experience-based foraging plasticity in mites: age dependency, primacy effects and memory persistence

**DOI:** 10.1098/rsos.172110

**Published:** 2018-04-18

**Authors:** Peter Schausberger, Undarmaa Davaasambuu, Stéphanie Saussure, Inga C. Christiansen

**Affiliations:** 1 Department of Behavioural Biology, University of Vienna, Althanstrasse 14, 1090 Vienna, Austria; 2 Group of Arthropod Ecology and Behavior, Department of Crop Sciences, University of Natural Resources and Life Sciences, Gregor Mendelstrasse 35, 1190 Vienna, Austria; 3 School of Agroecology, Mongolian University of Life Sciences, Ulaanbaatar 17024, Mongolia

**Keywords:** behavioural plasticity, early life experience, learning, predator–prey interactions

## Abstract

Behavioural plasticity can be categorized into activational (also termed contextual) and developmental plasticity. Activational plasticity allows immediate contextual behavioural changes, whereas developmental plasticity is characterized by time-lagged changes based on memory of previous experiences (learning). Behavioural plasticity tends to decline with age but whether this holds true for both plasticity categories and the effects of first-in-life experiences is poorly understood. We tackled this issue by assessing the foraging plasticity of plant-inhabiting predatory mites, *Amblyseius swirskii*, on thrips and spider mites following age-dependent prey experience, i.e. after hatching or after reaching maturity. Juvenile and young adult predator females were alternately presented thrips and spider mites, for establishing 1st and 2nd prey-in-life experiences, and tested, as gravid females, for their foraging plasticity when offered both prey species. Prey experience by juvenile predators resulted in clear learning effects, which were evident in likelier and earlier attacks on familiar prey, and higher proportional inclusion of familiar prey in total diet. First prey-in-life experience by juvenile but not adult predators resulted in primacy effects regarding attack latency. Prey experience by adult predators resulted mainly in prey-unspecific physiological changes, with easy-to-grasp spider mites providing higher net energy gains than difficult-to-grasp thrips. Prey experience by juvenile, but not adult, predators was adaptive, which was evident in a negative correlation between attack latencies and egg production. Overall, our study provides key evidence that similar experiences by juvenile and adult predators, including first-in-life experiences, may be associated with different types of behavioural plasticity, i.e. developmental and activational plasticity.

## 1. Introduction

Phenotypic plasticity of behaviour, i.e. the ability of the same genotype to flexibly express or develop a given behaviour, allows animals to adaptively cope with environmental variation and/or novelty [1–5]. Behavioural plasticity may be categorized into activational (also termed contextual) and developmental plasticity [3,5]. Activational plasticity refers to immediate behavioural changes, with the underlying neural network being already present; developmental plasticity refers to time-lagged behavioural changes following experience, because of the time needed to establish the underlying neural network and to consolidate memory of a given experience. Developmental plasticity of behaviour is typically dubbed learning, i.e. behavioural change based on experience. Developmental plasticity is more likely to occur, and to exert profound effects, during juvenile development, but is not restricted to the juvenile phase [1,3,6]. For example, among arthropods, holometabolous insects are commonly assumed to be better learners after reaching maturity than during juvenile development [7]. Developmental and activational plasticity may, but do not necessarily, operate independently. In many circumstances, especially in adult or age-advanced individuals whose developmental history and rearing history are unknown, allocating any observed behavioural plasticity to the developmental and activational categories is difficult (e.g. [8]). The reason is that behavioural plasticity expressed in advanced life phases may be the result, or an epiphenomenon, of developmental plasticity, i.e. learning, early in life (e.g. [5]). Thus, categorizing activational and developmental plasticity inevitably requires knowing about the developmental and rearing history of the experimental animals and their experiences.

We tackled this issue by comparing the behavioural plasticity of adult predatory mites *Amblyseius swirskii* in foraging contexts, following prey experiences made in the juvenile phase or made immediately after reaching maturity. *Amblyseius swirskii* is an omnivorous, plant-inhabiting predatory mite feeding on other mites, small insects such as thrips and whiteflies, and plant-derived substances such as pollen [9–12]. Its broad diet range makes it especially suitable for assessing foraging plasticity. Owing to its potential to feed on, and limit population growth of, significant horticultural pests such as western flower thrips *Frankliniella occidentalis*, two-spotted spider mite *Tetranychus urticae*, and tobacco whitefly *Bemisia tabaci*, *A. swirskii* has recently become an economically important, mass-reared natural enemy [13–15]. Like other predatory mites, e.g. *Phytoseiulus persimilis* and *Neoseiulus californicus* [16–18], *A. swirskii* can learn prey cues in early life, which improves foraging on this prey later in life [19,20]; its learning ability after reaching adulthood has not yet been assessed. *Amblyseius swirskii* develops from the egg to larva, protonymph, deutonymph to adult in about 7–8 days at 25°C [10]. The facultatively feeding larva and the obligatory feeding protonymph are highly sensitive to learning prey stimuli [19]. Prey experience in early life (thrips or spider mites) profoundly enhances the predation performance of *A. swirskii* on familiar prey after reaching adulthood, which is evident in shorter prey recognition times and attack latencies and higher predation rates [19–21]. The potential of *A. swirskii* to improve foraging by learning is higher for thrips than spider mites as prey [19,20]. The reason is that *T. urticae* is an easy-to-grasp prey, providing higher nutritional net gains/profitability to prey-naive predators, in terms of predator survival, developmental speed and reproduction, than the difficult-to-grasp *F. occidentalis* [9,10,12,22].

We investigated how previous experience with a given prey (two-spotted spider mites *T. urticae* and/or western flower thrips *F. occidentalis*), either during juvenile development or after reaching maturity, affects the foraging behaviour of adult *A. swirskii* females, as compared to prey-naive females. Additionally, we were interested in the influence of sequential experience with two prey species versus consistent experience with one prey species. Our primary objectives were (i) comparing the foraging plasticity following prey experiences during the juvenile and adult phase, (ii) examining primacy and persistence of memory of 1st prey experience in dependence of predator age (juvenile versus adult) and prey sequence, (iii) allocating the observed behavioural changes to the developmental and activational plasticity categories and (iv) elucidating the adaptive significance of foraging plasticity, i.e. its effects on reproductive success.

## 2. Methods

### 2.1. Experimental animals, population origins and rearing

*Amblyseius swirskii* used in the experiments derived from a laboratory-reared population founded with hundreds of specimens obtained from Koppert BV (Berkel en Rodenrijs, The Netherlands). The rearing units consisted of detached spider mite-infested primary leaves of common bean, *Phaseolus vulgaris*, placed upside down on a water-saturated foam cube and dusted with pollen of almond and maize, serving as food for the predators. Strips of moist tissue paper covered the cut petiole and edges of the leaves, to prevent the predators from escaping. Food was provided in 2- to 3-day intervals, by dusting pollen or by brushing spider mites from infested bean leaves onto the arena. Small cotton tufts under coverslips were placed on the arena to provide shelter and oviposition sites for the predators.

Prey used in the experiments were 1st larvae of *F. occidentalis* and nymphs of *T. urticae*. *Frankliniella occidentalis* was reared on detached leaves of *P. vulgaris* (approx. 11 × 13 cm) placed upside down on a 5% water agar solution in a closed Petri dish (14 cm in diameter). For ventilation, a circular opening (1 cm in diameter) was cut into the lid and covered with gauze. To obtain 1st larvae, adult thrips were randomly withdrawn from the stock population and transferred to a fresh bean leaf for 24 h for oviposition. After removing adult thrips, the Petri dish was stored in a climate chamber at 25 ± 1°C, 65 ± 5% relative humidity (RH) and 16 L : 8 D h photoperiod for 3.5 days. At that time most larvae had hatched and the Petri dish was kept in a fridge at 8°C and darkness to stop any further development of thrip larvae until use in experiments [19,20]. *Tetranychus urticae* was reared on whole common bean plants*.* Plants were grown at room temperature (23 ± 2°C) and 16 L : 8 D h photoperiod. For experiments, only proto- and deutonymphs were used as prey. The spider mites were manually brushed from infested leaves, using a paint brush, onto glass plates and then singly picked up and placed into experimental acrylic cages using a fine red marten's hair brush.

All rearing and (pre-)experimental units were kept in a climate chamber at 25 ± 1°C, 65 ± 5% RH and 16 L : 8 D photoperiod.

### 2.2. Pre-experimental procedures

To obtain even-aged eggs of *A. swirskii*, giving rise to experimental individuals, gravid females, recognizable by their expanded idiosomas, were randomly withdrawn from the stock population and placed on a fresh detached bean leaf arena harbouring spider mites and pollen. After 24 h, the predator eggs were collected, using a fine brush, and placed on a separate fresh leaf arena without any food (experiment 1) or with pollen of maize and almond (experiment 2). For experiment 1, the arena was checked for hatched larvae, to be used in the experiment, every 24 h. For experiment 2, the predators were left on the arena until reaching adulthood and mated females were used in the experiment.

### 2.3. Alternating prey experience by juvenile predators (experiment 1)

The first experiment aimed at examining the effects of alternating prey experiences during juvenile development on the foraging performance of adult predators. Cylindrical acrylic cages of 15 mm diameter and 3 mm height, laser-cut into rectangular acrylic plates, closed with fine gauze at the bottom and a microscope slide on the upper side, were used in the experiment. To start the prey experience phase (day 1), newly hatched predator larvae were picked from the leaf arena and singly placed inside acrylic cages, which had been previously loaded with either five spider mite nymphs (TU), three 1st larvae of thrips (FO) or three spider mite nymphs plus three 1st larvae of thrips (TU + FO), for 1 day. From the next day onwards (day 2; most predators were protonymphs by then), the predatory mites were transferred daily into a new cage containing either five spider mite nymphs or three 1st larvae of thrips until they reached adulthood (lasting 4–5 days; table 1). Daily prey supply was more than needed by the predators for optimal development. The sequence of using spider mites (TU) and/or thrips (FO) as 1st and/or 2nd prey during the prey experience phase resulted in six treatments: TU/TU, TU/FO, FO/TU, FO/FO, TU + FO/TU and TU + FO/FO (table 1). Each treatment was replicated 10–12 times. Inside the prey experience cages, the predatory mites could freely contact and feed on prey. After reaching adulthood, females were placed together with a male, randomly taken from the laboratory rearing, into a new cage for mating (day 7 or 8). One day later (day 8 or 9), the behavioural assay was started by transferring the gravid predatory mite female into a new cage containing six spider mite nymphs plus six 1st larvae of thrips. Every 5 to 10 min for 3 h, and again after 6 and 24 h, the cages were checked for prey killed and eaten by the predatory mite females. Attacks were scored successful when the predators had grasped prey and started to suck it out. The number of eggs laid by the predator females was counted 24 and 48 h after starting the behavioural assay.

**Table 1.** Predator life stages, and species and daily number of prey offered during the prey experience phase (1st and 2nd) and the behavioural assay of experiments 1 and 2. The number of prey items provided daily in the prey experience phase differed between experiments 1 and 2 because of different prey needs of juvenile and adult predators.

			prey experience phase		
	treatment	*N*	1st	2nd	behavioural assay
*experiment 1*					
day			1	2–7	8, 9
predator life stage^a^			L, P	P, D, A	A
prey^b^	TU/TU	11	5 TU	5 TU	6 TU + 6 FO
	TU/FO	11	5 TU	3 FO	6 TU + 6 FO
	FO/TU	10	3 FO	5 TU	6 TU + 6 FO
	FO/FO	9	3 FO	3 FO	6 TU + 6 FO
	TU + FO/TU	11	3 TU + 3 FO	5 TU	6 TU + 6 FO
	TU + FO/FO	12	3 TU + 3 FO	3 FO	6 TU + 6 FO
*experiment 2*					
day			1	2, 3	4–6
predator life stage^a^			A	A	A
prey^b^	TU/TU	12	12 TU	12 TU	6 TU + 6 FO
	TU/FO	12	12 TU	12 FO	6 TU + 6 FO
	FO/TU	11	12 FO	12 TU	6 TU + 6 FO
	FO/FO	12	12 FO	12 FO	6 TU + 6 FO
	TU + FO/TU	12	6 TU + 6 FO	12 TU	6 TU + 6 FO
	TU + FO/FO	12	6 TU + 6 FO	12 FO	6 TU + 6 FO

^a^L, larva; P, protonymph; D, deutonymph; A, adult female.

^b^TU, nymphs of spider mites, *T. urticae*; FO, 1st larvae of thrips, *F. occidentalis*.

### 2.4. Alternating prey experience by adult predators (experiment 2)

The second experiment aimed at examining the effects of alternating prey experiences on the foraging performance of adult predator females, which had been previously reared on a strictly vegetarian diet. To start the prey experience phase (lasting 3 days in total), gravid predatory mite females, which had been reared on leaf arenas with pollen for 10–12 days, were singly transferred into acrylic cages containing either 12 spider mite nymphs (TU), 12 1st larvae of thrips (FO) or six spider mite nymphs plus six 1st larvae of thrips (TU + FO) for 1 day. On day 2 and 3 of the prey experience phase, the females were transferred into a new cage containing either 12 spider mite nymphs or 12 1st larvae of thrips (table 1). In both phases, daily prey supply exceeded the daily prey needs of the predators. The number of prey items provided daily during the prey experience phase differed from experiment 1 because of higher prey needs of adult predators. On each day of the prey experience phase, the number of eggs laid by the predators was recorded. The sequence of using spider mites (TU) and/or thrips (FO) as 1st and/or 2nd prey during the prey experience phase resulted in six treatments: TU/TU, TU/FO, FO/TU, FO/FO, TU + FO/TU and TU + FO/FO (table 1). Each treatment was replicated 11–12 times. On day 4, the behavioural assay was started by transferring the predatory mite female into a new cage containing six spider mite nymphs plus six 1st larvae of thrips (table 1). The cages were checked for the time elapsed until successful attack on the 1st prey item in 15 min intervals over 3 h in total. First prey choice and attack were scored successful when the predators had grasped the prey and started to suck it out. On days 5 and 6, the females were transferred into new cages containing six spider mite nymphs plus six 1st larvae of thrips. On each day of the behavioural assay, the number of eggs laid and the number of prey eaten by the predators were recorded.

### 2.5. Statistical analyses

IBM SPSS Statistics for Windows, v. 23.0 (IBM Corp., Armonk, NY, USA) was used for all statistical analyses. All tests were two-sided.

In experiment 1, we used generalized estimating equations (GEE; normal distribution, identity link) to analyse the influence of the 1st and 2nd prey during the prey experience phase on the proportion of *T. urticae* killed (arcsine square-root transformed before analysis) within 3 h (maximum of three items counted) and 6 h of the behavioural assay (time used as auto-correlated within-subject variable). GEE (binomial distribution, log link) was used to examine the influence of the 1st and 2nd prey during the prey experience phase and prey rank (1st, 2nd and 3rd prey chosen; used as auto-correlated within-subject variable) on the species identity of the first three prey items. Generalized linear models (GLM; gamma distribution, log link) were used to analyse the attack latency of *A. swirskii*, first, as affected by the 1st chosen prey, and, second, as affected by the 1st and 2nd prey during the prey experience phase within each 1st chosen prey, *T. urticae* and *F. occidentalis.* GEE (gamma distribution, log link) was used to analyse the influence of the 1st and 2nd prey during the prey experience phase on the number of eggs produced by *A. swirskii* within 24 and 48 h (time used as within-subject variable) of the behavioural assay. Nonlinear (logarithmic) regression was used to analyse the relationship between attack latency and the number of eggs produced within 48 h.

In experiment 2, we used GEE (normal distribution, identity link) to analyse the influence of the 1st and 2nd prey during the prey experience phase on the proportion of *T. urticae* killed (arcsine square-root transformed before analysis) within 24, 48 and 72 h of the behavioural assay (time used as auto-correlated within-subject variable). Separate GLMs were used to examine the influence of the 1st and 2nd prey during the prey experience phase on the 1st chosen prey species (binomial distribution, log link) and the attack latency on *T. urticae* and *F. occidentalis* (gamma distribution, identity link). GEE (Poisson distribution, log link) was used to analyse the influence of the 1st and 2nd prey during the prey experience phase on the number of eggs produced by *A. swirskii* during the prey experience phase and the behavioural assay (used as auto-correlated within-subject variable with exchangeable structure). Curve estimation in regression analysis was used to analyse the relationship between attack latency and the number of eggs produced during the behavioural assay.

## 3. Results

### 3.1. Alternating prey experience by juvenile predators (experiment 1)

**Figure 1.**
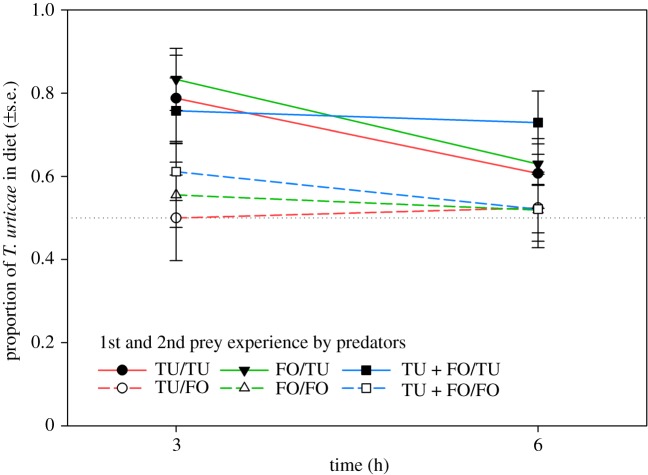
Mean proportion of *T. urticae* in diet of adult *A. swirskii* females offered six nymphs of *T. urticae* (TU) and six 1st larvae of *F. occidentalis* (FO) after 3 and 6 h of the behavioural assay (experiment 1). During the preceding prey experience phase, the juvenile females received in their larval and early protonymphal stage either TU or FO or both, TU + FO, as 1st prey, and from the late protonymphal stage until adulthood either TU or FO as 2nd prey (table 1). Predators that had experienced TU as 2nd prey (solid lines) included a higher proportion of TU in diet than predators that had experienced FO as 2nd prey (broken lines) (GEE; *p* < 0.001). The dotted reference line represents random expectation in the binary choice situation.

**Figure 2.**
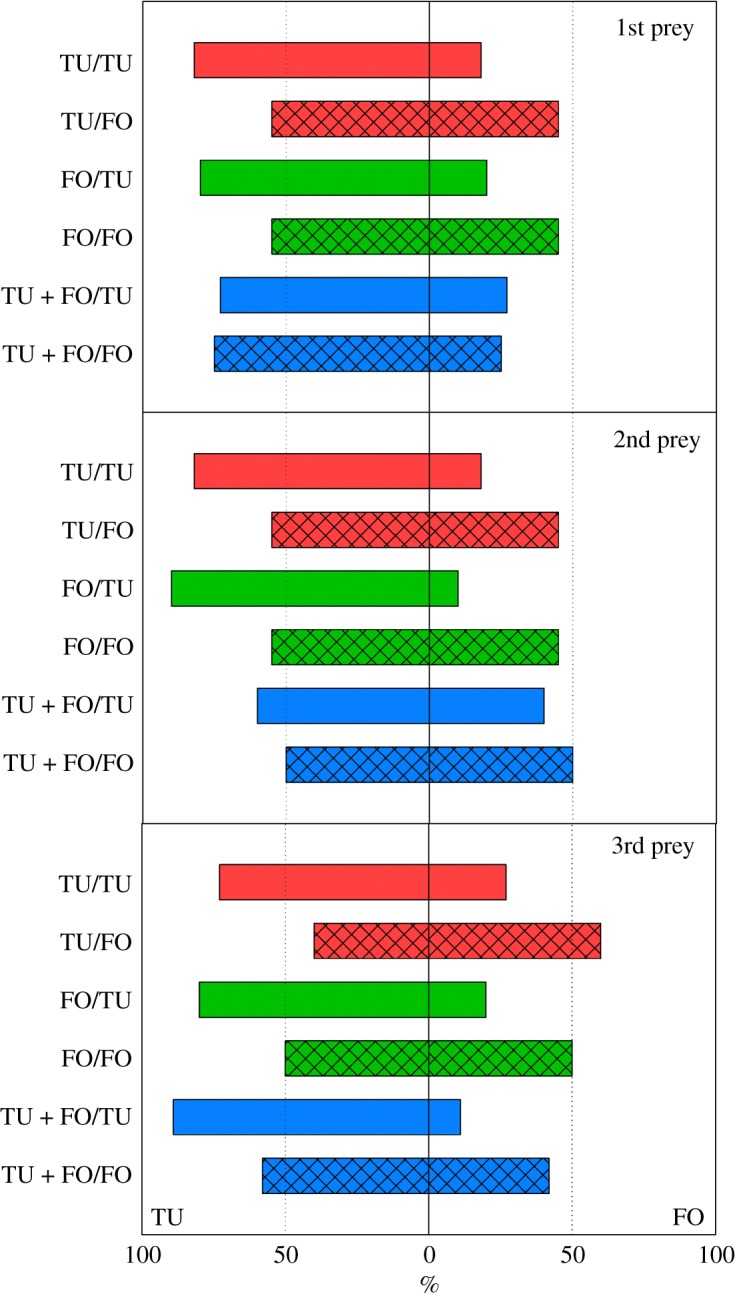
Percentage of adult *A. swirskii* females, offered six nymphs of *T. urticae* (TU) and six 1st larvae of *F. occidentalis* (FO), choosing either TU or FO as 1st, 2nd and 3rd prey during the behavioural assay (experiment 1). During the preceding prey experience phase, the juvenile females received in their larval and early protonymphal stage either TU or FO or both, TU + FO, as 1st prey, and from the late protonymphal stage until adulthood either TU or FO as 2nd prey (table 1). Sequential prey choice by predators that had experienced TU as 2nd prey (bars with uniform fill) was more strongly biased towards TU than sequential prey choice by predators that had experienced FO as 2nd prey (bars with grid fill) (GEE; *p* < 0.001). The dotted reference lines represent random expectation in the binary choice situation.

**Figure 3.**
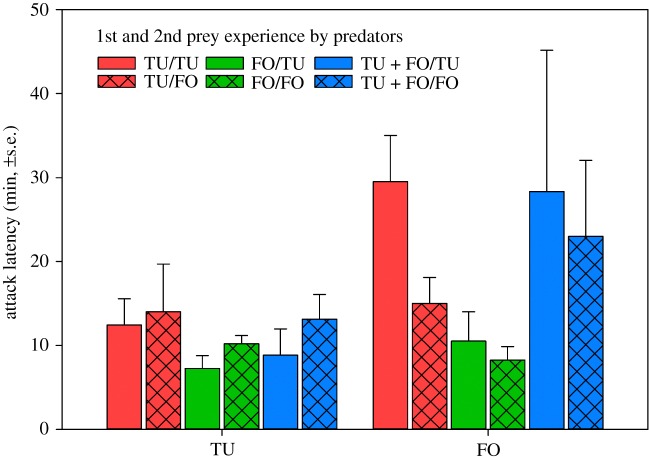
Mean attack latency on the 1st prey item by adult *A. swirskii* females offered a choice of six nymphs of *T. urticae* (TU) and six 1st larvae of *F. occidentalis* (FO) in the behavioural assay (experiment 1). During the preceding prey experience phase, the juvenile females received in their larval and early protonymphal stage either TU or FO or both, TU + FO, as 1st prey, and from the late protonymphal stage until adulthood either TU or FO as 2nd prey (table 1). Predators first attacking TU attacked earlier than predators first attacking FO (GLM; *p* < 0.001). Among predators first attacking FO, predators that had experienced FO as 1st prey in life (green bars) attacked earlier than predators that had experienced TU or TU + FO as 1st prey in life (red and blue bars) (LSD following GLM; *p* < 0.05).

**Figure 4.**
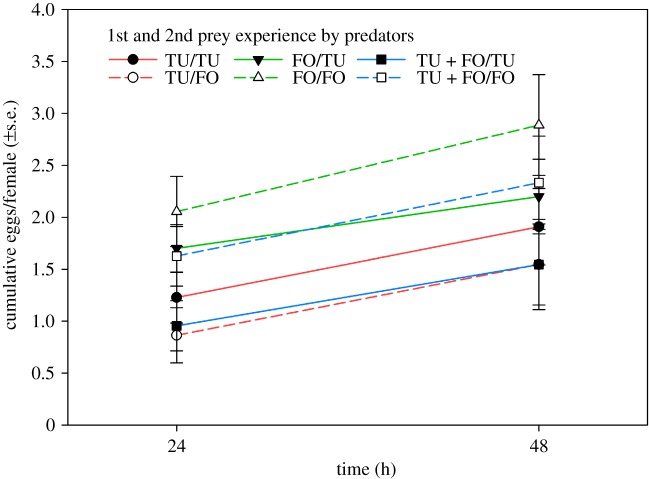
Cumulative mean number of eggs produced within 24 and 48 h by adult *A. swirskii* females offered six nymphs of *T. urticae* (TU) and six 1st larvae of *F. occidentalis* (FO) in the behavioural assay (experiment 1). During the preceding prey experience phase, the juvenile females received in their larval and early protonymphal stage either TU or FO or both, TU + FO, as 1st prey, and from the late protonymphal stage until adulthood either TU or FO as 2nd prey (table 1). Predators that had experienced FO as 1st prey in life (green lines) produced more eggs than predators that had experienced TU or TU + FO as 1st prey in life (red and blue lines) (LSD following GLM; *p* < 0.05).

**Figure 5.**
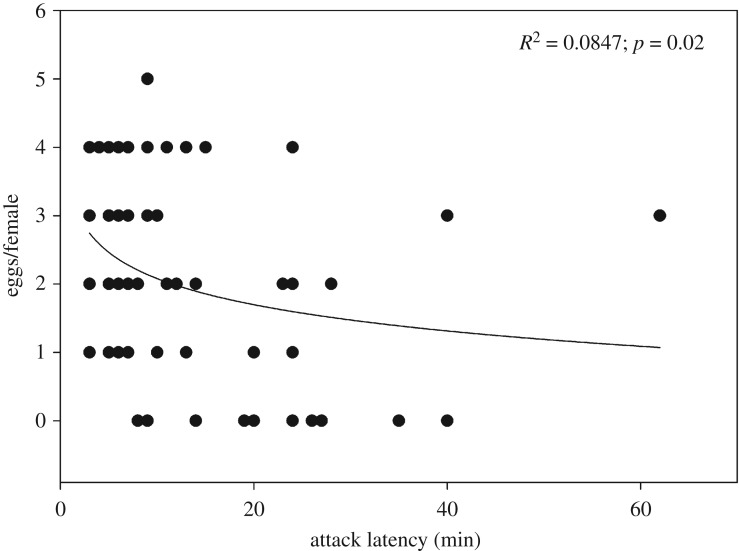
Number of eggs produced within 48 h regressed (logarithmic; *y* = *a* + *b* × ln(*x*)) on the attack latency by adult *A. swirskii* females offered six nymphs of *T. urticae* (TU) and six 1st larvae of *F. occidentalis* (FO) in the behavioural assay (experiment 1). During the preceding prey experience phase, the juvenile females received in their larval and early protonymphal stage either TU or FO or both, TU + FO, as 1st prey, and from the late protonymphal stage until adulthood either TU or FO as 2nd prey (table 1).

The proportion of *T. urticae* in diet during the initial 3 and 6 h of the behavioural assay (figure 1) was higher if the 2nd prey during development was *T. urticae* (GEE; Wald $\chi_{1}^{2}=10.171,$ *p* < 0.001), but was not influenced by the 1st prey (Wald $\chi_{2}^{2}=0.649,$ *p* = 0.72) and the interaction between 1st and 2nd prey (Wald $\chi_{2}^{2}=0.004,$ *p* = 0.99). Similarly, sequential prey choice (figure 2) was more strongly biased towards *T. urticae* in predators experiencing *T. urticae* as 2nd prey during development, relative to those experiencing *F. occidentalis* (GEE; Wald $\chi_{1}^{2}=10.307,$ *p* < 0.001), but was unaffected by the 1st prey (Wald $\chi_{2}^{2}=0.270,$ *p* = 0.87) and the interaction between 1st and 2nd prey (Wald $\chi_{2}^{2}=1.631,$ *p* = 0.44). Pooled across treatments, predators choosing first *T. urticae* attacked earlier than predators choosing first *F. occidentalis* (GLM; Wald $\chi_{1}^{2}=838.197,$ *p* < 0.001) (figure 3). The attack latency of predators choosing first *T. urticae* (figure 3) was unaffected by the 1st prey during development (Wald $\chi_{2}^{2}=3.488,$ *p* = 0.18), the 2nd prey (Wald $\chi_{1}^{2}=1.857,$ *p* = 0.17) and the interaction between 1st and 2nd prey (Wald $\chi_{2}^{2}=0.256,$ *p* = 0.88). By contrast, the attack latency of predators choosing first *F. occidentalis* was influenced by the 1st prey during development (Wald $\chi_{2}^{2}=22.433,$ *p* < 0.001) but not by the 2nd prey (Wald $\chi_{1}^{2}=2.675,$ *p* = 0.10) and the interaction between 1st and 2nd prey (Wald $\chi_{2}^{2}=1.651,$ *p* = 0.44); predators first experiencing *F. occidentalis* were faster in attacking *F. occidentalis* than those first experiencing *T. urticae* or both prey species (LSD; *p* < 0.05) (figure 3). Egg production (figure 4) was influenced by the 1st prey during development (Wald $\chi_{2}^{2}=9.672,$ *p* = 0.008) but not by the 2nd prey (Wald $\chi_{1}^{2}=0.992,$ *p* = 0.32) and the interaction between 1st and 2nd prey (Wald $\chi_{2}^{2}=4.415,$ *p* = 0.11); predators first experiencing *F. occidentalis* produced more eggs than those first experiencing *T. urticae* or both prey species (LSD; *p* < 0.05). The number of eggs produced within 48 h was negatively correlated with the attack latency (logarithmic regression; *R*² = 0.085, *F*_1,61_ = 5.642, *p* = 0.02) (figure 5).

### 3.2. Alternating prey experience by adult predators (experiment 2)

**Figure 6.**
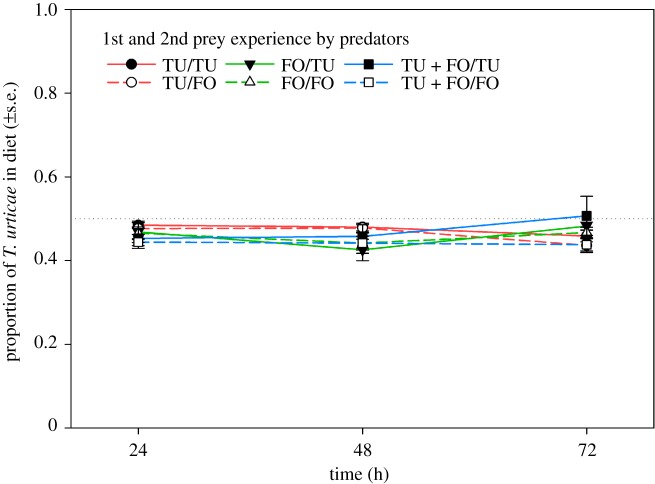
Mean proportion of *T. urticae* in diet of adult *A. swirskii* females offered six nymphs of *T. urticae* (TU) and six 1st larvae of *F. occidentalis* (FO) over 3 days of the behavioural assay (experiment 2). During the preceding prey experience phase, the adult females received either TU or FO or both, TU + FO, as 1st prey, and either TU or FO as 2nd prey (table 1). During juvenile development, the females were exclusively fed on pollen, i.e. were prey-naive until entering the prey experience phase. 1st and 2nd prey experience did not have any significant effects (GEE; *p* > 0.05). The dotted reference line represents random expectation in the binary choice situation.

**Figure 7.**
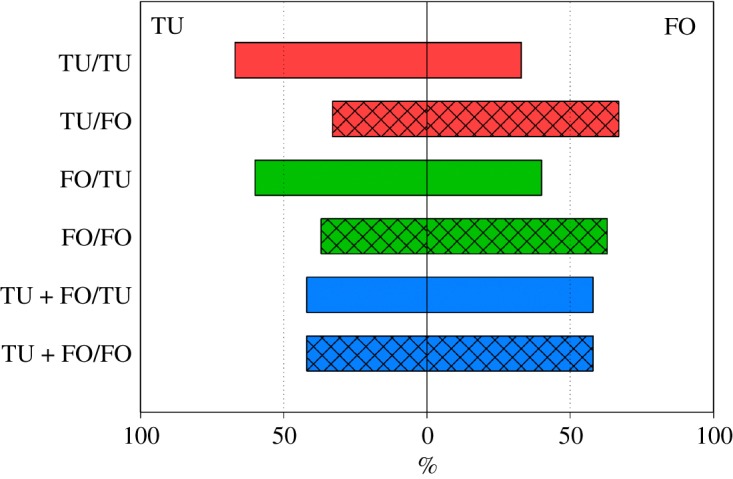
Percentage of adult *A. swirskii* females, offered six nymphs of *T. urticae* (TU) and six 1st larvae of *F. occidentalis* (FO), choosing either TU or FO as 1st prey in the behavioural assay (experiment 2). During the preceding prey experience phase, the adult females received either TU or FO or both, TU + FO, as 1st prey, and either TU or FO as 2nd prey (table 1). During juvenile development the females were exclusively fed on pollen, i.e. were prey-naive until entering the prey experience phase. 1st and 2nd prey experience did not have any significant effects (GEE; *p* > 0.05). The dotted reference lines represent random expectation in the binary choice situation.

**Figure 8.**
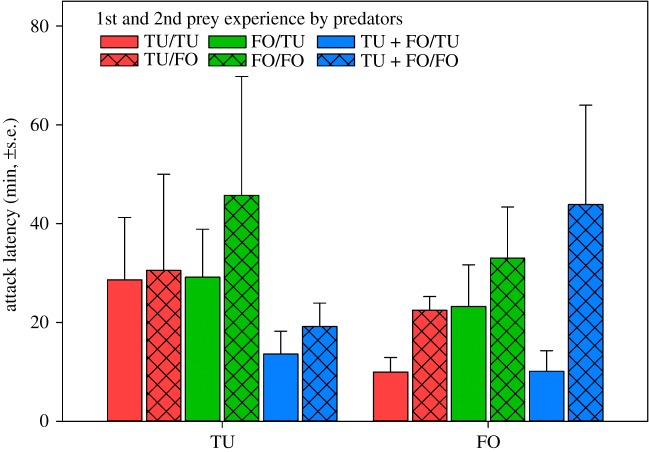
Mean attack latency on the 1st prey chosen by *A. swirskii* females offered six nymphs of *T. urticae* (TU) and six 1st larvae of *F. occidentalis* (FO) in the behavioural assay (experiment 2). During the preceding prey experience phase, the adult females received either TU or FO or both, TU + FO, as 1st prey, and either TU or FO as 2nd prey (table 1). During juvenile development, the females were exclusively fed on pollen, i.e. were prey-naive until entering the prey experience phase. Across treatments, attack latencies on TU and FO were similar (GLM; *p* = 0.25). Predators that had experienced FO as 2nd prey (bars with grid fill) attacked later than predators that had experienced TU as 2nd prey (bars with uniform fill) (GLM; *p* = 0.005).

**Figure 9.**
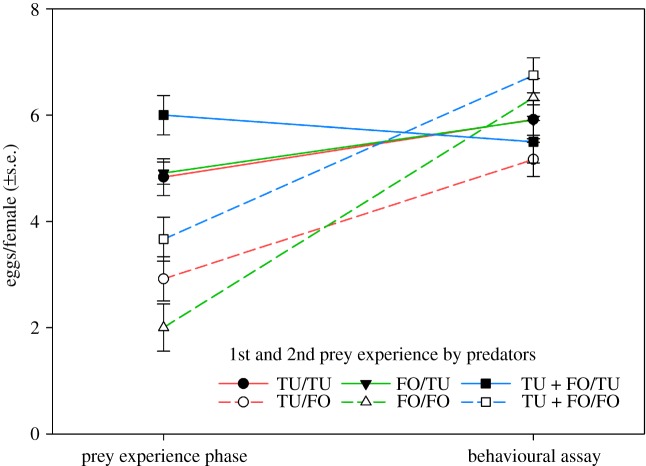
Mean number of eggs produced by *A. swirskii* females either receiving *T. urticae* (TU) or *F. occidentalis* (FO) or both (TU + FO) as 1st prey, and either TU or FO as 2nd prey during the 3 days experience phase, and then offered six TU nymphs and six 1st larvae of FO during the 3 days behavioural assay (experiment 2; table 1). During juvenile development, the females were exclusively fed on pollen, i.e. were prey-naive until entering the prey experience phase. Egg production during the behavioural assay was higher than egg production during the prey experience phase (GEE; *p* < 0.001). During the prey experience phase, but not during the behavioural assay, predators experiencing TU as 2nd prey (solid lines) produced more eggs than predators experiencing FO as 2nd prey (broken lines) (GEE; *p* < 0.001).

**Figure 10.**
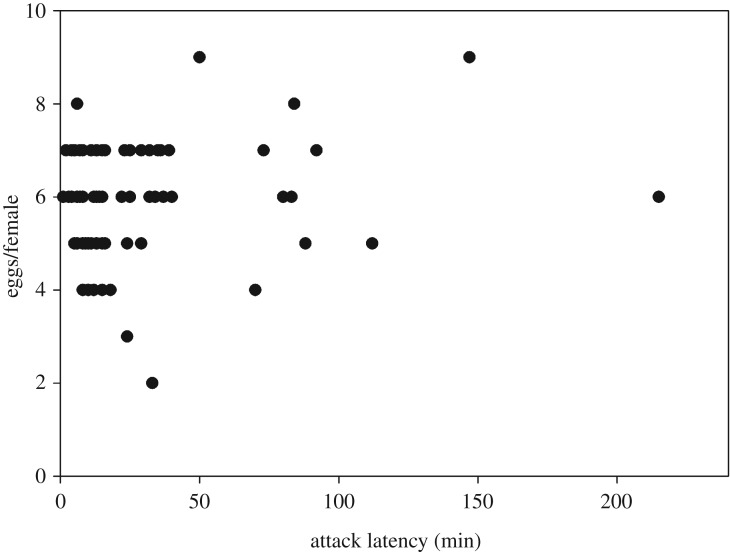
Number of eggs produced during the 3 days behavioural assay regressed on attack latency by *A. swirskii* offered six nymphs of *T. urticae* (TU) and six 1st larvae of *F. occidentalis* (FO) (experiment 2). During the preceding prey experience phase, the adult females received either TU or FO or both, TU + FO, as 1st prey, and either TU or FO as 2nd prey (table 1). During juvenile development, the females were exclusively fed on pollen, i.e. were prey-naive until entering the prey experience phase. Curve estimation (linear, logarithmic, cubic, quadratic, logistic, exponential regression) did not reveal any significant relationship between attack latency and number of eggs produced (*p* > 0.05).

The proportion of *T. urticae* in diet during the behavioural assay was unaffected by the 1st (GEE; Wald $\chi_{2}^{2}=1.810,$ *p* = 0.40) and 2nd prey during the experience phase (Wald $\chi_{1}^{2}=2.735,$ *p* = 0.10) and their interaction (Wald $\chi_{2}^{2}=3.021,$ *p* = 0.22) (figure 6). Similarly, 1st prey choice in the behavioural assay was independent of the 1st (GLM; Wald $\chi_{2}^{2}=0.319,$ *p* = 0.85) and 2nd prey (GLM; Wald $\chi_{1}^{2}=2.772,$ *p* = 0.10) during the experience phase and their interaction (Wald $\chi_{2}^{2}=1.504,$ *p* = 0.47) (figure 7). Across treatments, *T. urticae* and *F. occidentalis* were similarly early attacked (GLM; Wald $\chi_{1}^{2}=1.314,$ *p* = 0.25) (figure 8). The 2nd (Wald $\chi_{1}^{2}=7.981,$ *p* = 0.005) but not 1st (Wald $\chi_{2}^{2}=4.284,$ *p* = 0.12) prey during the experience phase influenced the attack latency; predators that had experienced *F. occidentalis* as 2nd prey attacked later than those that had experienced *T. urticae* as 2nd prey (figure 8). None of the pairwise interactions had a significant effect on attack latency (*p* > 0.12 for each). The number of eggs produced was higher during the behavioural assay than during the prey experience phase (GEE; Wald $\chi_{1}^{2}=29.767,$ *p* < 0.001) and higher in predators experiencing *T. urticae* as 2nd prey (Wald $\chi_{1}^{2}=13.485,$ *p* < 0.001) (figure 9). However, the latter was only true in the prey experience phase but not the behavioural assay, as indicated by the significant interaction (Wald $\chi_{1}^{2}=17.547,$ *p* < 0.001). The 1st prey during the experience phase had a marginally significant influence on egg production (Wald $\chi_{2}^{2}=5.146,$ *p* = 0.08); predators first experiencing both prey species produced in total slightly more eggs than predators first experiencing either *T. urticae* or *F. occidentalis* (figure 9). Curve estimations did not indicate any significant relationship between the attack latency and the number of eggs produced during the behavioural assay (*R*² < 0.06, *F* < 1.964, *p* > 0.16 for linear, logarithmic, cubic, quadratic, logistic, exponential regressions) (figure 10).

## 4. Discussion

Prime objectives in interpreting the results of our experiments were (i) attributing the observed behavioural variations to purely physiological effects, arising from a more favourable physiological state/higher vigour, due to previous prey providing higher net energy gains, and/or to learning, i.e. memory of previous prey experiences, and (ii) allocating the observed behavioural plasticities to the developmental and activational categories [3,5]. Basically, we assume that mere changes in physiological state (vigour, nourishment) should have resulted in prey-unspecific effects (positive or negative) in predation success and oviposition during the behavioural assay, whereas learning should have resulted in prey-specific enhancement of foraging. Owing to thrips intrinsically being a more difficult-to-get prey for prey-naive predators than spider mites, learning the cues of thrips entails a greater potential for *A. swirskii* to enhance foraging than learning the cues of spider mites [19,20]. In experiment 2, use of prey-naive individuals—prey-naive because of exclusive rearing on pollen during juvenile development—excludes that any observed behavioural plasticity represents an epiphenomenon of prey experience-based plasticity developed in the juvenile phase [5,8].

Based on the above considerations, we argue that prey experiences made in the juvenile phase (experiment 1) have long-lasting, profound learning effects on the foraging behaviour of adult predatory mite females *A. swirskii*, whereas those made by prey-naive predators after reaching maturity (experiment 2) produce mainly physiological, but negligible learning, effects. Primacy effects of 1st prey-in-life experiences were only apparent if the 1st prey-in-life experience occurred in the juvenile phase (experiment 1). Memorizing the 1st prey-in-life experience by adult predatory mites in the behavioural assay was only evident when this experience was made early in the juvenile phase but not when it was made after reaching maturity. The occurrence of sensitive periods early in life, and behavioural plasticity declining with advancing age, is known from numerous animals (for review, see [7]). Experiencing the difficult prey *F. occidentalis* by juvenile predators (particularly, if 1st prey in life) produced more apparent learning effects than experiencing the easy prey *T. urticae* (experiment 1). Prey experiences made in the juvenile phase resulted in a time-lagged change in behaviour, qualifying as experience-based developmental plasticity (experiment 1). By contrast, prey experiences made after reaching maturity mainly affected the predators' physiological state. Experiment 2 did not provide any indication that predators lacking prey experience during the juvenile phase maintained or prolonged their sensitive periods, as observed in other animals deprived of informative cues early in life (for review, see [7]). Accordingly, foraging plasticity following prey experiences made by adult predators can be largely categorized as activational plasticity (experiment 2). Juvenile experience-based developmental plasticity was adaptive, which was evident in learning effects (memory of first thrips prey experience allowing shorter attack latencies on thrips) correlating with higher egg production (experiment 1). First prey experience of juvenile predators on the difficult prey *F. occidentalis*, but not the easy prey *T. urticae*, adaptively improved energy allocation after reaching maturity, as indicated by higher egg production (see also [19]). By contrast, egg production by adult *A. swirskii* females lacking prey experiences during the juvenile phase was determined by the immediate prey ingested; egg production during the behavioural assay did not correlate with any effects of previous prey experiences made after reaching maturity (experiment 2).

Regarding attack latency in experiment 1, the 2nd prey experience of juvenile predators did not override their 1st prey experience. By contrast, 2nd but not 1st prey experience of juvenile predators influenced proportional prey ingestion; predators experiencing *T. urticae* as 2nd prey included a higher proportion of *T. urticae* in their diet than predators experiencing *F. occidentalis* as 2nd prey. Shifts in proportional inclusion of thrips and spider mites in diet were prey-specific and biased towards familiar prey, pointing at experience-based developmental plasticity, i.e. learning [19–21]. Xu & Enkegaard [23] observed that, in choice situations, adult *A. swirskii* females preferred thrips over spider mites if they had not experienced either of those two prey species during the juvenile phase. This was similar to our observations in experiment 2, where, across treatments, the predators likelier first attacked thrips than spider mites. Experiment 1 suggests that adult *A. swirskii* females, which experienced one or both prey species during the juvenile phase, do not have a preference for thrips but tend to first go for spider mites. Comparing initial prey choice and proportional predation on *F. occidentalis* and *T. urticae* between experiments 1 and 2 suggests that prey experience in the juvenile phase may induce shifts in the predators’ relative preference for these two prey species.

The results of experiment 2 suggest mainly physiological effects of the 2nd prey during the experience phase and activational plasticity. In experiment 2, 1st prey-in-life experience did not have any effect on 1st prey choice, attack latency and predation rate during the behavioural assay. Predation in the behavioural assay was mainly determined by the 2nd prey during the experience phase but these effects were prey species-unspecific and apparently primarily due to changes in physiological state (vigour; nourishment) but not learning. Nutritionally more favourable spider mites as 2nd prey allowed the predators to un-specifically shorten their attack latencies. Lack of 2nd prey experience-based learning was especially apparent in longer attack latencies on thrips by predator females receiving thrips as 2nd prey than by predator females receiving spider mites as 2nd prey. When starting the behavioural assay, predators feeding on thrips as 2nd prey during the experience phase were obviously in poorer physiological condition than those offered spider mites as 2nd prey. Feeding on thrips providing lower net energy gains was also evident in that predators receiving thrips as 2nd prey in the experience phase produced fewer eggs in this phase than predators receiving spider mites as 2nd prey.

Overall, our study provides a key example of age-dependent operation of developmental and activational foraging plasticity [1,3,7]. Prey experiences during the juvenile phase mostly resulted in developmental plasticity, whereas prey experiences of adult predators had mainly physiological effects. Foraging plasticity of adult predators that had only experienced prey in the adult but not juvenile phase thus represented activational plasticity. The findings of our study have great relevance for the use of *A. swirskii* in biological control. Mass rearing protocols should provide for target pest experiences by juvenile predators, to increase their efficacy as biocontrol agents. Release of target pest-experienced predators should provide a decisive head start in predator population growth and pest suppression if target pest-naive predators suffer from deficiencies in developmental plasticity arising from rearing on factitious food.

## Supplementary Material

Raw data of the experiments

## Data accessibility

The datasets supporting this article have been uploaded as part of the electronic supplementary material.

## References

[1] Mery F, Burns JG 2010 Behavioral plasticity: an interaction between evolution and experience. Evol. Ecol. 24, 571–583. (doi:10.1007/s10682-009-9336-y)

[2] Bateson P, Gluckman P 2011 Plasticity, robustness, development and evolution. Cambridge, UK: Cambridge University Press.

[3] Snell-Rood EC 2013 An overview of the evolutionary causes and consequences of behavioural plasticity. Anim. Behav. 85, 1004–1011. (doi:10.1016/j.anbehav.2012.12.031)

[4] Snell-Rood EC, Davidowitz G, Papaj DR 2013 Plasticity in learning causes immediate and trans-generational changes in allocation of resources. Integrat. Comp. Biol. 53, 329–339. (doi:10.1093/icb/ict030) 10.1093/icb/ict030 23624867

[5] Stamps JA 2016 Individual differences in behavioural plasticities. Biol. Rev. 91, 534–567. (doi:10.1111/brv.12186) 25865135 10.1111/brv.12186

[6] Stamps JA, Krishnan VV 2017 Age-dependent changes in behavioural plasticity: insights from Bayesian models of development. Anim. Behav. 126, 53–67. (doi:10.1016/j.anbehav.2017.01.013)

[7] Papaj DR, Lewis AC 1993 Insect learning. Ecological and evolutionary perspectives. New York, NY: Chapman & Hall.

[8] Hesselberg T 2014 The mechanism behind plasticity of web-building behavior in an orb spider facing spatial constraints. J. Arachnol. 42, 311–314. (doi:10.1636/J14-05.1)

[9] Momen FM, El Saway SA 1993 Biology and feeding behavior of the predatory mite, *Amblyseius swirskii* (Acari, Phytoseiidae). Acarologia 34, 199–204.

[10] Wimmer D, Hoffmann D, Schausberger P 2008 Prey suitability of western flower thrips, *Frankliniella occidentalis*, and onion thrips, *Thrips tabaci*, for the predatory mite *Amblyseius swirskii*. Biocont. Sci. Technol. 18, 533–542. (doi:10.1080/09583150802029784)

[11] Goleva I, Zebitz CPW 2013 Suitability of different pollen as alternative food for the predatory mite *Amblyseius swirskii* (Acari, Phytoseiidae). Exp. Appl. Acarol. 61, 259–283. (doi:10.1007/s10493-013-9700-z) 23670826 10.1007/s10493-013-9700-z

[12] Riahi E, Fathipour Y, Talebi AA, Mehrabadi M 2017 Linking life table and consumption rate of *Amblyseius swirskii* (Acari: Phytoseiidae) in presence and absence of different pollens. Ann. Entomol. Soc. Am. 110, 244–253. 10.1093/jee/tox172 28854656

[13] Nomikou M, Janssen A, Schraag R, Sabelis MW 2001 Phytoseiid predators as potential biological control agents for *Bemisia tabaci*. Exp. Appl. Acarol. 25, 271–291. (doi:10.1023/A:1017976725685) 11603735 10.1023/a:1017976725685

[14] Messelink GJ, van Maanen R, van Steenpaal SEF, Janssen A 2008 Biological control of thrips and whiteflies by a shared predator: two pests are better than one. Biol. Control 44, 372–379. (doi:10.1016/j.biocontrol.2007.10.017)

[15] Arthurs S, McKenzie CL, Chen J, Dogramaci M, Brennan M, Houben K, Osborne L 2009 Evaluation of *Neoseiulus cucumeris* and *Amblyseius swirskii* (Acari: Phytoseiidae) as biological control agents of chilli thrips, *Scirtothrips dorsalis* (Thysanoptera: Thripidae) on pepper. Biol. Control 49, 91–96. (doi:10.1016/j.biocontrol.2009.01.002)

[16] Schausberger P, Walzer A, Hoffmann D, Rahmani H 2010 Food imprinting revisited: early learning in foraging predatory mites. Behaviour 147, 883–897. (doi:10.1163/000579510X495799)

[17] Rahmani H, Hoffmann D, Walzer A, Schausberger P 2009 Adaptive learning in the foraging behavior of the predatory mite *Phytoseiulus persimilis*. Behav. Ecol. 20, 946–950. (doi:10.1093/beheco/arp081)

[18] Schausberger P, Peneder S 2017 Non-associative versus associative learning by foraging predatory mites. BMC Ecol. 17, 2 (doi:10.1186/s12898-016-0112-x) 28088215 10.1186/s12898-016-0112-x PMC5237478

[19] Christiansen IC, Szin S, Schausberger P 2016 Benefit-cost trade-offs of early learning in foraging predatory mites *Amblyseius swirskii*. Sci. Rep. 6, 23571 (doi:10.1038/srep23571) 27006149 10.1038/srep23571 PMC4804281

[20] Seiter M, Schausberger P 2016 Constitutive and operational variation of learning in foraging predatory mites. PLoS ONE 11, e0166334 (doi:10.1371/journal.pone.0166334) 27814380 10.1371/journal.pone.0166334 PMC5096697

[21] Reichert MB, Christiansen IC, Seiter M, Schausberger P 2017 Transgenerational loss and recovery of early learning ability in foraging predatory mites. Exp. Appl. Acarol. 71, 243–258. (doi:10.1007/s10493-017-0122-1) 28409405 10.1007/s10493-017-0122-1 PMC5403862

[22] Buitenhuis R, Shipp L, Scott-Dupree C 2010 Intra-guild vs. extra-guild prey: effect on predator fitness and preference of *Amblyseius swirskii* (Athias-Henriot) and *Neoseiulus cucumeris* (Oudemans) (Acari: Phytoseiidae). Bull. Entomol. Res. 100, 167–173. (doi:10.1017/S0007485309006944) 19419591 10.1017/S0007485309006944

[23] Xu X, Enkegaard A 2010 Prey preference of the predatory mite, *Amblyseius swirskii* between first instar western flower thrips *Frankliniella occidentalis* and nymphs of the two-spotted spider mite *Tetranychus urticae*. J. Insect Sci. 10, 149 (doi:10.1673/031.010.14109) 21070175 10.1673/031.010.14109 PMC3016914

